# Effectiveness of nurse-led mHealth-based self-care management for patients with heart failure: a systematic review and meta-analysis

**DOI:** 10.3389/fcvm.2026.1882692

**Published:** 2026-09-01

**Authors:** Ting Wu, Ying Ying Jia, Ruo Han Fu, Shuang Zou, Jian Ping Song

**Affiliations:** 1 Nursing Department, The Second Affiliated Hospital of Zhejiang University School of Medicine, Hangzhou, China; 2 Zhejiang University School of Medicine, Hangzhou, China; 3 Nursing Department, Sir Run Run Shaw Hospital, Zhejiang University School of Medicine, Hangzhou, China

**Keywords:** heart failure, meta-analysis, mHealth, self-management, systematic review

## Abstract

**Aims:**

The aim of this study was to systematically review the evidence for the effectiveness of mHealth self-care management in patients with Heart Failure (HF).

**Design:**

Systematic review and meta-analysis.

**Data sources:**

A comprehensive search of PubMed, Embase, the Cochrane Library, Web of Science, CINAHL, CNKI, CBM, Wan fang, and VIP databases from February 1, 2025 to January 1, 2026.

**Methods:**

This study included only randomized controlled trials (RCTs) that compared the effects of mHealth interventions with usual care in adult patients with HF. The revised Cochrane risk-of-bias tool for randomized trials (RoB2) was used to assess the risk of bias for each study. The Grading of Recommendations, Assessment, Development, and Evaluation (GRADE) criteria were used to rate the certainty of the evidence. Meta-analysis was performed using Review Manager (RevMan v.5.4) and R software.

**Results:**

In total, 28 RCTs met the inclusion criteria. Compared with the usual-care group, nurse-led mHealth self-management interventions could significantly reduce HF-related readmissions [odds ratio (OR) 0.61, 95% CI 0.48–0.78, *P* < 0.0001] and all-cause readmissions (OR 0.53, 95% CI 0.41–0.69, *P* < 0.00001), but no significant differences were observed in mortality. The meta-analyses also showed that nurse-led mHealth interventions could improve self-care behaviors and quality of life, increase patients’ knowledge of HF and medication adherence, and enhance cardiac function. However, there was no significant improvement in the mental status of the nurse-led mHealth intervention group compared with the control group.

**Conclusion:**

Nurse-led mHealth self-management interventions can benefit the health of patients with heart failure in multiple ways. However, the clinical effects are influenced by various factors, and more high-quality studies are needed to confirm their effectiveness.

**Systematic Review Registration:**

PROSPERO CRD420251005175, https://www.crd.york.ac.uk/PROSPERO/view/CRD420251005175.

## 1 Introduction

Heart failure (HF) represents the end stage of most cardiovascular diseases and remains a major contributor to morbidity and mortality worldwide (1, 2). It is estimated that 64.3 million people worldwide are living with HF, with a global prevalence ranging from 1% to 3% of the general population (3, 4). With population ageing and the increasing prevalence of cardiovascular risk factors, the burden of HF continues to rise, with evidence suggesting a shift toward a younger age at onset (5). HF can seriously affect patients' physical function and health-related quality of life, and is associated with psychological distress such as depression and anxiety (6), resulting in a substantial global disease burden (7).

Multiple lines of evidence have indicated that self-management is an effective way to improve the outcomes of chronic diseases, and it is also a core element of contemporary heart failure care (8, 9). Nurses are central to primary health care and are often the first point of contact across clinical settings. In heart failure management, nurses often deliver essential components of disease management and serve as an indispensable part of the healthcare team (10). Many systematic reviews published in recent years have shown that nurse-led interventions can improve clinical and patient-reported outcomes, demonstrating the crucial role of nursing in patients' self-management (11, 12).

In the past few years, with the advancement of mobile communication technologies and the widespread use of mobile devices, mobile health (mHealth) has developed rapidly, ushering the management of patients with heart failure into the era of information technology (13, 14). In a broad sense, mHealth is defined as the application of mobile computing and wireless communication technologies in healthcare (15). It can extend nursing services beyond the hospital setting and support patients in self-monitoring and self-management. Nurse-led mHealth refers to the provision of health services and health information by nurses to the population through tools such as the Internet, smartphones, and mobile applications, helping patients develop healthy lifestyle behaviors (16). The emergence of mHealth has provided a timely means to support the long-term management needs of patients with heart failure (17).

A growing body of evidence suggests that mHealth self-management interventions can improve outcomes in a range of chronic diseases (18, 19). However, research specifically evaluating nurse-led mHealth self-management remains limited. Although nurse-led mHealth self-management interventions have been applied in patients with HF (20, 21), conclusions across existing systematic reviews are not fully consistent, likely due to heterogeneity in care-delivery models, intervention components and intensity, outcome definitions and measurement, and other methodological differences (22, 23). In addition, a rigorous and comprehensive synthesis of randomized evidence is still lacking, particularly with respect to key clinical and patient-reported outcomes.

To address these research gaps, this systematic review aims to characterize the current intervention strategies of nurse-led mHealth programs for patients with HF and better understand its potential benefits and limitations, to evaluate their effectiveness compared with standard treatment or usual care, and to elucidate the effects of nurse-led mHealth interventions on outcomes in this population.

## 2 Methods

The current study was a retrospective analysis exclusively on published research material, with no involvement of human subjects. Therefore, the Institution Review Board approval was deemed unnecessary based on the institutional policies. This systematic review protocol was registered with PROSPERO (ID: CRD420251005175) and follows the methodology of the Preferred Reporting Items for Systematic Reviews and Meta-Analyses (PRISMA) guidelines (24).

### 2.1 Literature search strategy

The search strategy was developed with support from an experienced medical information specialist. Two graduate students (TW and YYJ), both trained in evidence-based methods, independently conducted the systematic searches to identify eligible studies. A comprehensive literature search was performed on the following databases: PubMed, Embase, the Cochrane Library, Web of Science, Cumulative Index to Nursing and Allied Health Literature (CINAHL), China National Knowledge Infrastructure (CNKI), China Biological Medicine (CBM), Wan fang database, and Wei Pu (VIP), to identify all pertinent articles. This search aimed to systematically review the evidence for the effectiveness of mHealth self-care management in patients with HF. Retrieval time limit ranged from the corresponding database's inception until February 1, 2025, and updated till January 1, 2026. A combination of Mesh terms and free-text words was employed in the literature search. The following search terms were used: “Heart Failure,” “Cardiac Failure,” “Heart Decompensation,” “Telemedicine,” “Telenursing,” “Digital Health,” “Self-Management,” “Self-Care,” “Self-Medication,” and other related terms.

### 2.2 Literature inclusion and exclusion criteria

The population, intervention, comparison, outcome, and study design (PICOS) question was as follows: Among adult patients with heart failure (P), do nurse-led mHealth-based self-care management interventions (I), compared with usual care or standard treatment (C), improve clinical outcomes and patient-reported outcomes, including readmission, mortality, self-care behaviors, quality of life, heart failure knowledge, medication adherence, exercise capacity, and mental status (O), in randomized controlled trials (S)? This PICOS question guided the eligibility criteria for study selection (25). The inclusion criteria were as follow: (1) Adults (age ≥18 years of age) who had been diagnosed with HF; (2) the interventions were nurse-led mHealth-based self-care management programs that incorporated at least one mHealth component (e.g., mobile applications). Educational content was delivered via internet/web-based programs accessible on smartphones, tablets, or other mobile devices, as well as through SMS messaging and social media-based education. Nurses provided at least 50% of the intervention in terms of the frequency or duration of care delivery; (3) the comparison was HF patients who received standard treatment or usual care; and the (4) outcomes included primary and secondary outcomes. Primary outcomes are directly related to the disease, including all-cause readmission rate, all-cause mortality rate, HF readmission rate, cardiovascular mortality rate and self-care behaviors. The secondary outcomes comprised quality of life, HF knowledge, medication adherence, the 6-minute walk test, and mental status. (5) Only randomized controlled trials were included. No language and date restrictions were set.

Literature exclusion criteria were as follows: studies were excluded if the full text was unavailable, if they were duplicate or overlapping publications, or if they were reviews, integrative evidence studies, conference abstracts, editorials, research protocols, or cross-sectional studies conducted during the early stages of scale development.

### 2.3 Study selection and data extraction

Following the literature search, retrieved records were imported into EndNote X9 (Clarivate) and duplicates were removed. Two reviewers independently screened titles and abstracts and subsequently assessed full-text eligibility. During the procedures, any differences between the two researchers were resolved through dialogue or by consulting a third researcher (RHF). Any discrepancies were resolved through discussion until consensus was reached.

The following characteristics were extracted from all eligible studies: name of the first author, publication year, country, descriptions of the control, and the intervention, participant characteristics (age, sex, cardiac function classification, and left ventricular ejection fraction), type of mHealth technologies, target of mHealth intervention, duration of intervention, Characteristics of mHealth interventions, recruitment setting, and outcomes. All outcome data included in the meta-analysis were extracted independently by the same two reviewers (TW and YYJ). Data charting and result synthesis were also conducted independently by two researchers (TW and YYJ).

### 2.4 Risk of bias assessment and quality of evidence

Two reviewers (TW and RHF) independently assessed the risk of bias for all studies using RoB2 tool (26), and consulted the third reviewer for their opinion when necessary. This tool assesses 5 domains to address different types of bias: randomization process, deviations from the intended interventions, missing outcome data, measurement of the outcome, and selection of the reported result. All studies were ranked in five different domains yielding results of low risk of bias, some concerns of bias, or high risk of bias. Evaluation of evidence certainty for each outcome was performed using the GRADE approach (27). This approach rates the risk of bias, inconsistency, indirectness, imprecision, and other considerations as “high,” “moderate,” “low,” or “very low”. The GRADE assessment was completed with the GRADE pro Guideline Development Tool.

### 2.5 Statistical analysis

A meta-analysis was conducted using Review Manager version 5.4.1 (The Cochrane Collaboration) and illustrated using a forest plot when at least 2 studies were measured for the same outcomes for heart failure at the longest follow-up timepoint (28). Relative risk (RR) is used for count data, and mean difference (MD) is used for measurement data, both with 95% confidence intervals (CI). Heterogeneity was assessed using the *I^2^* statistic and *P* value (29). A fixed-effect model was used when *P* ≥ 0.10 and *I^2^* ≤ 50%, indicating no substantial heterogeneity; otherwise, a random-effects model was applied when *P* < 0.10 and *I^2^* > 50%. Sensitivity analyses were performed using a leave-one-out approach to assess the robustness of pooled results for outcomes with substantial heterogeneity (30). When the number of included studies is ≥10, we assessed publication bias using funnel plots and the Egger test (31) in R software.

## 3 Results

### 3.1 Search outcomes

**Figure 1:**
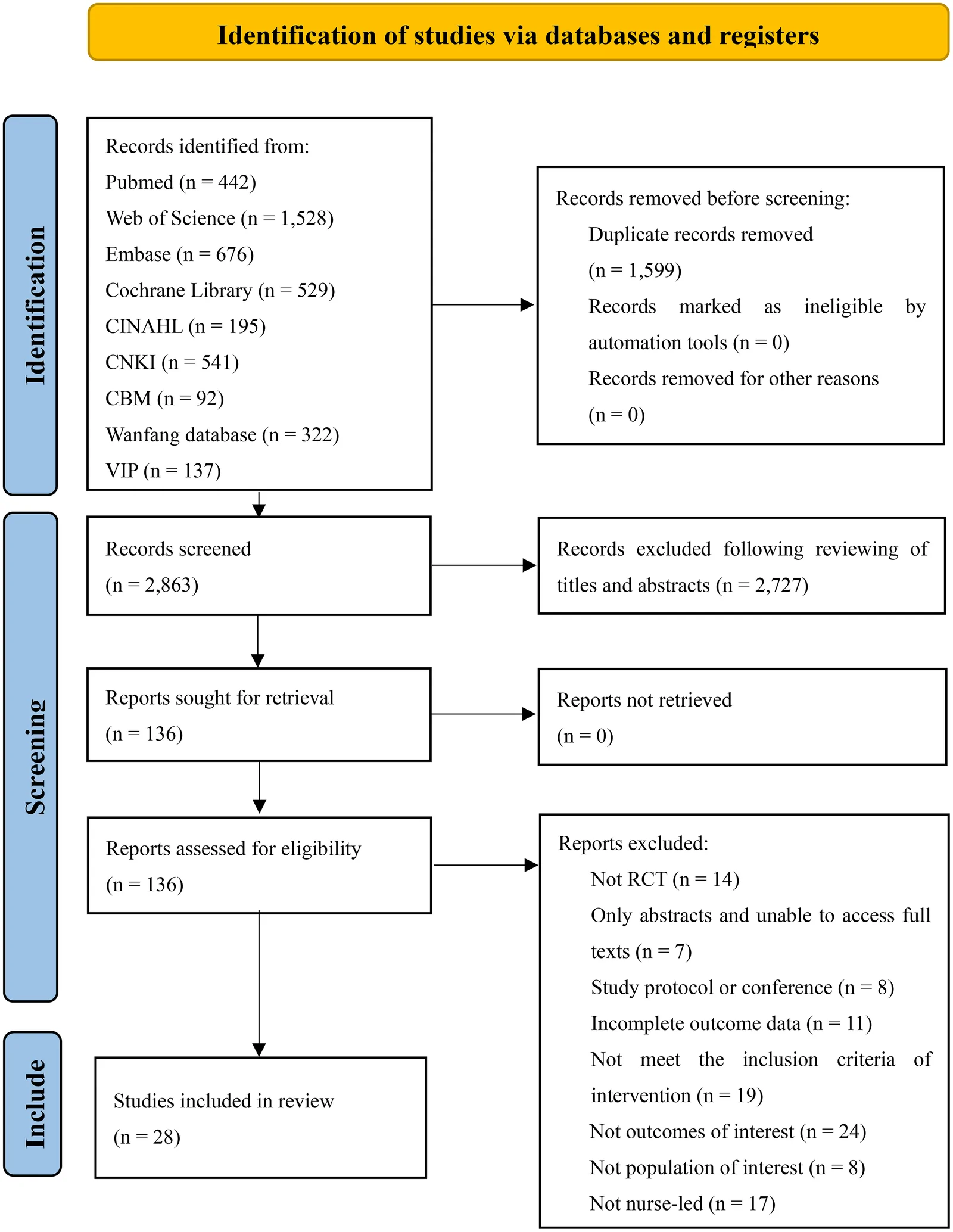
PRISMA 2020 flowchart.

A total of 4,462 articles were retrieved in the literature search. After removal of 1,599 duplicates, 2,863 titles and abstracts were screened in relation to the inclusion/exclusion criteria. Of these, 2,727 articles were excluded and a total of 136 articles were subject to full-text review. Finally, 28 RCTs meeting the inclusion criteria were included in the systematic review and meta-analysis (Figure 1).

### 3.2 Risk of bias and quality assessment

**Figure 2:**
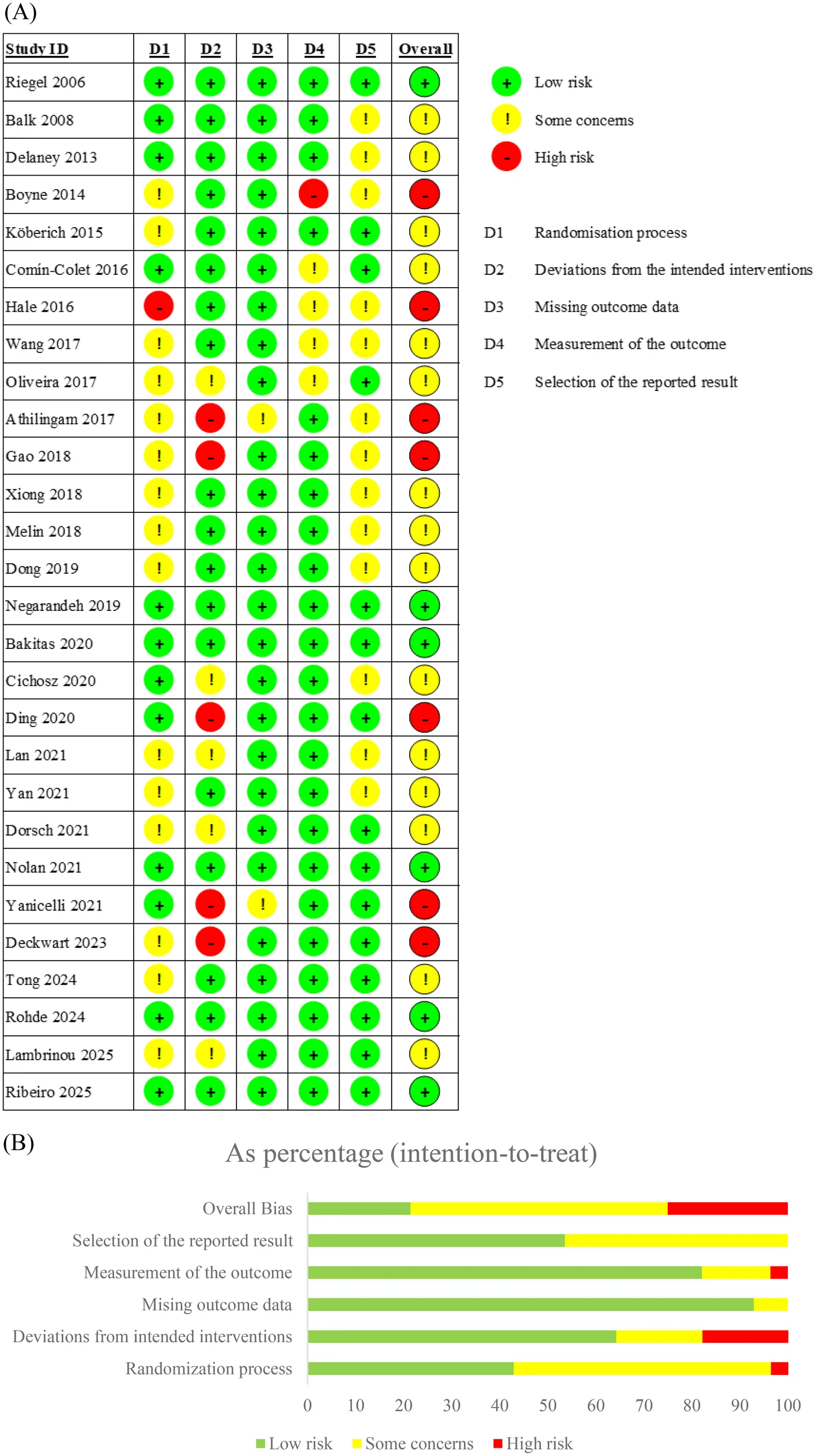
Quality assessment. **(A)** Each risk of bias domain for each included study. **(B)** Each risk of bias domain presented as a percentage across all included studies.

The results of risk of bias assessment are summarized in Figure 2. For the randomization process, 12 studies were judged as having a low risk of bias, 15 studies were judged to have some concerns due to insufficient reporting of random sequence generation or allocation concealment, and 1 study was judged as high risk due to potential discrepancies in baseline data (32). Owing to the nature of the interventions, the studies could not have been blinded to participants and 5 studies were judged to have some concerns regarding deviations from the intended interventions. Five studies were determined as “high risk” for the category of measurement of the outcome because all questionnaire scores were patient-reported, which may have possibly affected the outcomes. Regarding missing outcome data, the vast majority of studies were judged to be at low risk of bias (26 studies), with only 2 studies raising some concerns. Most studies utilized intention-to-treat analysis, reliable measures, and suitable statistical analyses, and only 1 study was judged as high risk because outcomes relied exclusively on patient-reported questionnaire scores, which may have increased susceptibility to measurement bias (33). In addition, almost all studies were registered on registration platforms and reported all outcomes. Among the 28 included studies, the overall risk of bias was assessed as low in 6 studies (21.4%), as having some concerns in 15 studies (53.6%), and as high in 7 studies (25%).

### 3.3 Characteristics of included studies

A total of 28 articles published between 2006 and 2025 were included, comprising 5,882 participants with heart failure. Overall, 2,928 patients were allocated to the mHealth intervention group and 2,954 to the usual-care group. The studies were conducted across 14 countries, with the largest number undertaken in China (7 studies), followed by the United States (5 studies). The mean age of the participants ranged from 33 to 90 years. In terms of gender distribution, the proportion of female patients with heart failure in the mHealth groups was 17.2% to 67.2%, and that in the usual care groups was 6.7% to 70%. The New York Heart Association (NYHA) Functional Classification categorizes patients into four classes according to the extent of their limitations in physical activity. Relevant data on this classification were reported in 26 studies, with the enrolled participants falling into NYHA Class I to Class IV. Most trials were single-center (15/28), whereas the remaining 13 randomized controlled trials (RCTs) were multicenter studies. The basic characteristics of the included studies are presented in Table 1, whereas the characteristics of participants and details of interventions are summarized in Supplementary Material Table S1.

**Table 1:** Basic characteristics of studies included in the meta-analysis.

Author (year)	Country	NYHA Grade	Type of mHealth technologies	Target of mHealth intervention	Settings
Riegel et al. (21) (2006)	Mexico	Ⅱ-Ⅳ	Telephone	Education	2 participating community hospitals close to the US-Mexico border
Balk et al. (34) (2008)	Netherlands	Ⅰ-Ⅳ	Designed telemedical system (MOTIVA system)	Monitoring+Education	8 Dutch hospitals (one heart failure/transplant centre, seven general hospitals)
Delaney et al. (35) (2013)	United States	Ⅲ-Ⅳ	Designed telemedical system (The HomMed Health Monitor)	Monitoring+Education	a large multibranch home care agency in Connecticut
Boyne et al. (33) (2014)	Netherlands	Ⅱ-Ⅳ	Designed telemedical system (Health Buddy)	Monitoring+Education	Maastricht University Medical Centre, Atrium Medical Center, Orbis Medical Concern
Köberich et al. (36) (2015)	Germany	Ⅱ-Ⅳ	Telephone	Education	2 sites of a university-afﬁliated medical centre in southern Germany
Comín-Colet et al. (37) (2016)	Spain	Ⅰ-Ⅳ	Designed telemedical system (The home Tele-HealthCare platform)	Monitoring+Education	Hospital del Mar
Hale et al. (32) (2016)	United States	Ⅰ-Ⅲ	Designed telemedical system (The MedSentry medication monitoring system)	Monitoring+Reminders	Massachusetts General Hospital (MGH), Brigham and Women’s Hospital (BWH)
Author (year)	Country	NYHA Grade	Type of mHealth technologies	Target of mHealth intervention	Settings
Wang et al. (38) (2017)	China	Ⅱ-Ⅳ	Mobile or tablet app (WeChat)	Monitoring+Education	Beijing Shi Jitan Hospital Affiliated to Capital Medical University
Oliveira et al. (39) (2017)	Brazil	Ⅰ-Ⅲ	Telephone	Education	Hospital Universitário Pedro Ernesto - HUPE
Athilingam et al. (40) (2017)	United States	Ⅰ-Ⅲ	Designed telemedical system (HeartMapp)	Monitoring+Education	A tertiary hospital affiliated with the University of South Florida
Gao et al. (41) (2018)	China	Ⅰ-Ⅳ	Mobile or tablet app (WeChat)	Education	Affiliated Hospital of Xuzhou Medical University
Xiong et al. (42) (2018)	China	NA	Designed telemedical system (Youdeyi)	Education	The Second People’s Hospital of Guangdong Province
Melin et al. (43) (2018)	Sweden	Ⅱ-Ⅲ	Mobile or tablet app (OPTILOGG)	Monitoring+Education	Karolinska University Hospital​​, Danderyd University Hospital, Södersjukhuset
Dong et al. (44) (2019)	China	Ⅱ-Ⅳ	Designed telemedical system	Monitoring+Education	Zhongshan Hospital Affiliated to Fudan University
Negarandeh et al. (45) (2019)	Iran	Ⅱ-Ⅲ	Telephone	Education	Grand Hospital of Dezful University of Medical Sciences
Bakitas et al. (46) (2020)	United States	Ⅲ-Ⅳ	Telephone	Education	University of Alabama at Birmingham, Birmingham Veterans Affairs Medical Center
Cichosz et al. (47) (2020)	Denmark	Ⅱ-Ⅲ	Designed telemedical system	Monitoring	All 11 municipalities in the region and Aalborg University
Ding et al. (48) (2020)	Australia	Ⅰ-Ⅲ	Designed telemedical system	Monitoring+Reminders	Peninsula Health in Victoria, Fiona Stanley Hospitale in Western Australia
Lan et al. (49) (2021)	China	Ⅱ-Ⅳ	Designed telemedical system (Heart Care Manager)	Monitoring+Education	People’s Hospital of Guangxi Zhuang Autonomous Region
Yan et al. (50) (2021)	China	Ⅱ-Ⅳ	Mobile or tablet app (WeChat)	Monitoring+Reminders	Zhejiang Hospital
Dorsch et al. (51) (2021)	United States	Ⅰ-Ⅳ	Mobile or tablet app (ManageHF4Life)	Monitoring	Michigan Medicine, The University of Michigan’s academic medical center
Nolan et al. (52) (2021)	Canada	Ⅰ-Ⅲ	Designed telemedical system (telephone, web and e-mail)	Monitoring+Education	The heart function clinics of 3 tertiary care hospitals in Toronto, Vancouver, and Ottawa
Yanicelli et al. (53) (2021)	Argentina	Ⅰ-Ⅳ	Designed telemedical system (HTS)	Monitoring+Education	Zenon Santillan Health Center Hospital
Deckwart et al. (54) (2023)	Germany	Ⅰ-Ⅳ	Designed telemedical system	Monitoring	No information
Tong et al. (55) (2024)	China	NA	Designed telemedical system	Monitoring+Education	A Grade-A Tertiary Hospital in Henan Province
Rohde et al. (56) (2024)	Brazil	Ⅰ-Ⅳ	Mobile text message	Monitoring+Education	5 geographical regions in Brazil
Lambrinou et al. (57) (2025)	The Republic of Cyprus	Ⅰ-Ⅳ	Telephone	Education	The four (biggest) public hospitals in the Republic of Cyprus
Ribeiro (20) (2025)	Brazil	Ⅲ-Ⅳ	Telephone and Mobile text message	Monitoring+Education	6 public hospitals in Belo Horizonte

### 3.4 Intervention characteristics

The programs were performed by specialty nurses (*n* = 8), registered nurses (*n* = 7), advanced practice nurses (*n* = 3), nurse case managers (*n* = 3), research nurses (*n* = 2), or individuals with expertise in more than one health-related discipline (*n* = 5). These studies used various types of mHealth technologies for the intervention (Table 1). Of these, 15 studies used a designed telemedical system, 6 studies used the telephone, 5 studies used a mobile phone or tablet app, 1 study used mobile text messages, and 1 study used a combination of telephone with mobile text messages. The duration of the intervention ranged from 1 month to 12 months (17 studies had interventions lasting ≥6 months).

### 3.5 Primary outcomes

#### 3.5.1 Effects of mHealth interventions on HF-related readmission

**Figure 3:**
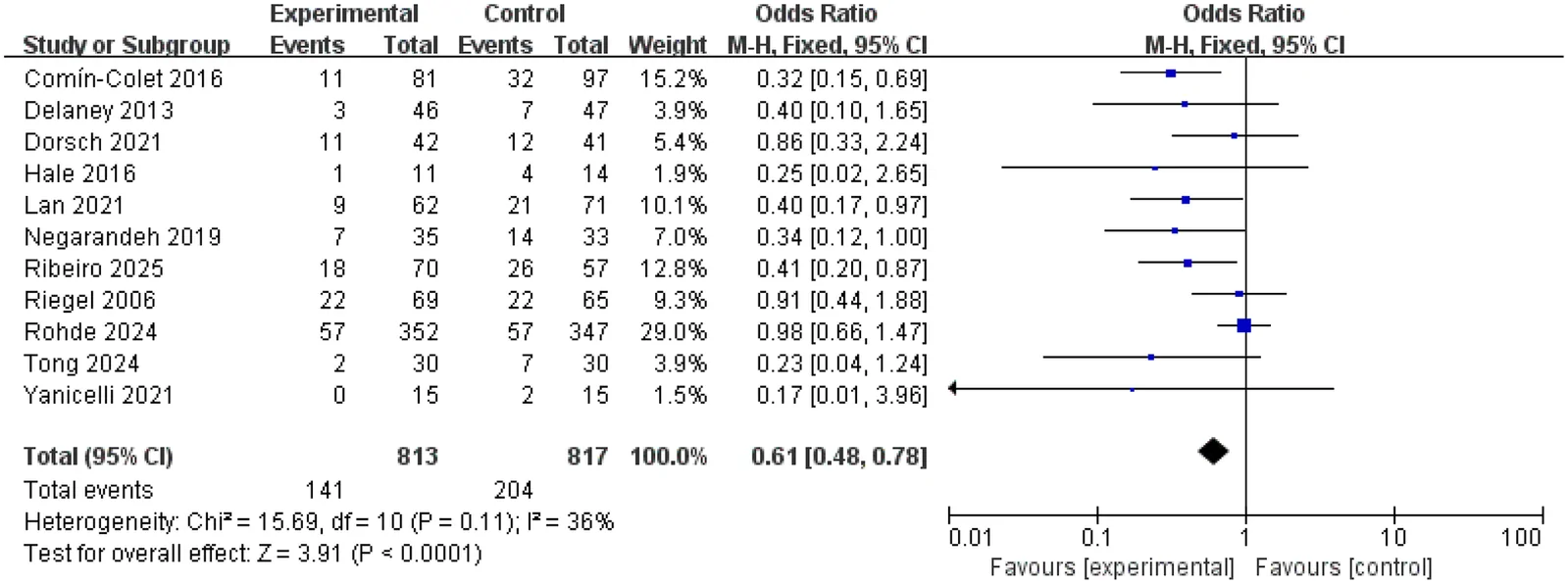
Forest plot of the effects of mHealth interventions on HF-related readmission

A total of 11 studies reported HF-related readmission data. The 11 studies included 1,630 participants and had moderate heterogeneity (*P* = 0.11; *I^2^* = 36%). The analysis showed that HF-related readmission was significantly lower in the nurse-led mHealth intervention group compared with the control group (OR 0.61, 95% CI 0.48–0.78, *P* < 0.0001) (Figure 3).

#### 3.5.2 Effects of mHealth interventions on all-cause readmission

**Figure 4:**
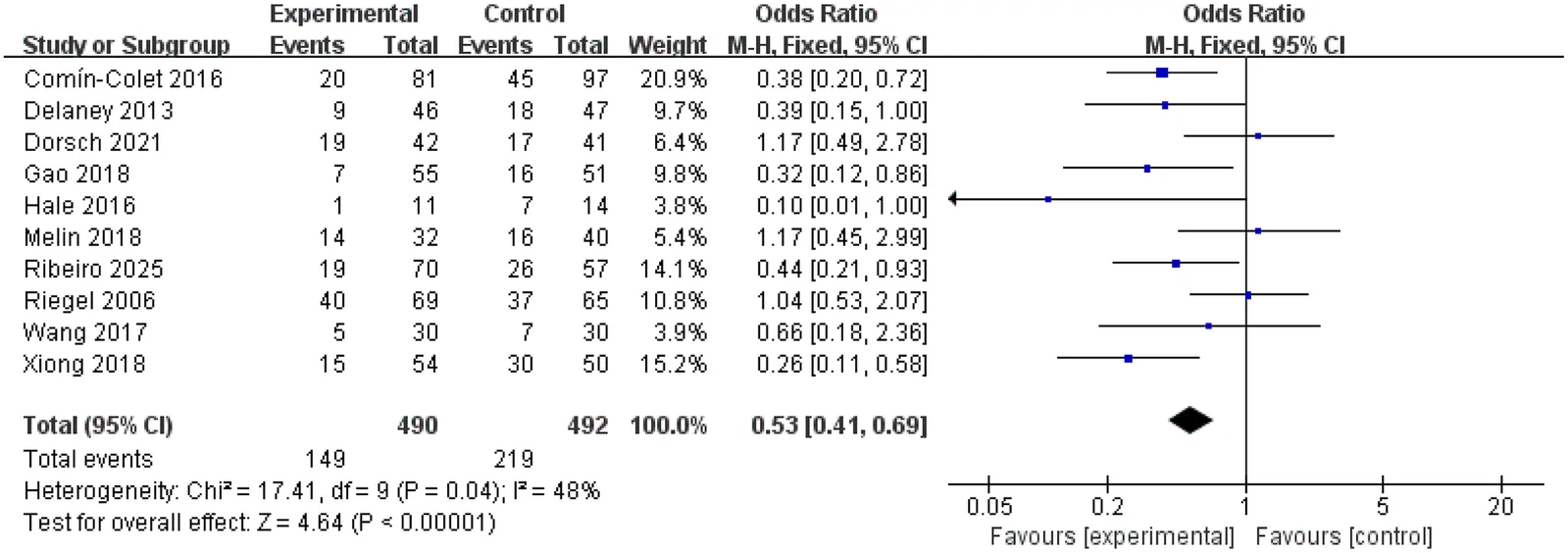
forest plot of the effects of mHealth interventions on all-cause readmission.

A total of 10 studies reported all-cause readmission data. The 10 studies included 982 participants and had moderate heterogeneity (*P* = 0.04; *I^2^* = 48%). The analysis showed that all-cause readmission was significantly lower in the nurse-led mHealth intervention group compared with the control group (OR 0.53, 95% CI 0.41–0.69, *P* < 0.00001) (Figure 4).

#### 3.5.3 Effects of mHealth interventions on cardiovascular mortality

**Figure 5:**
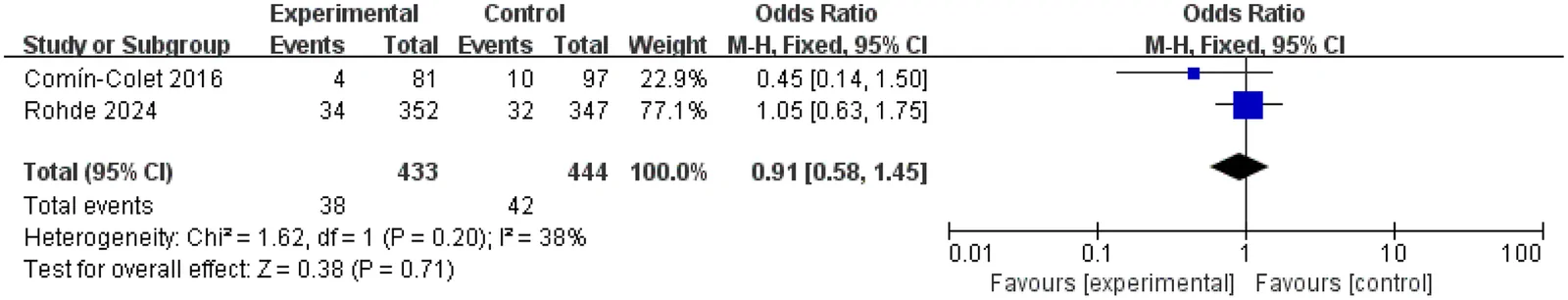
Forest plot of the effects of mHealth interventions on cardiovascular mortality.

Only 2 studies reported cardiovascular mortality data. The 2 studies included 877 participants and had moderate heterogeneity (*P* = 0.20; *I^2^* = 38%). The analysis showed no significant difference in cardiovascular mortality between the nurse-led mHealth intervention group and the usual-care group. (OR 0.91, 95% CI 0.58–1.45, *P* = 0.71) (Figure 5).

#### 3.5.4 Effects of mHealth interventions on all-cause mortality

**Figure 6:**
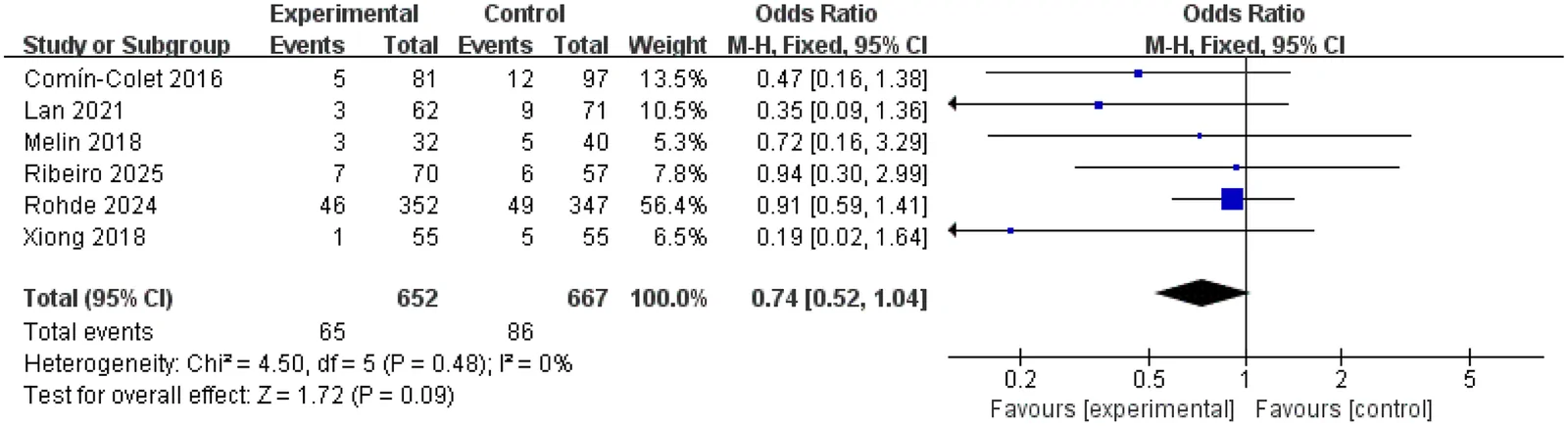
Forest plot of the effects of mHealth interventions on all-cause mortality.

A total of 6 studies reported all-cause mortality data. The 6 studies included 1,319 participants and had low heterogeneity (*P* = 0.48; *I^2^* = 0%). The analysis showed no significant difference in all-cause mortality between the nurse-led mHealth intervention group and the usual-care group. (OR 0.74, 95% CI 0.52–1.04, *P* = 0.09) (Figure 6).

#### 3.5.5 Effects of mHealth interventions on self-care behaviors

**Figure 7:**
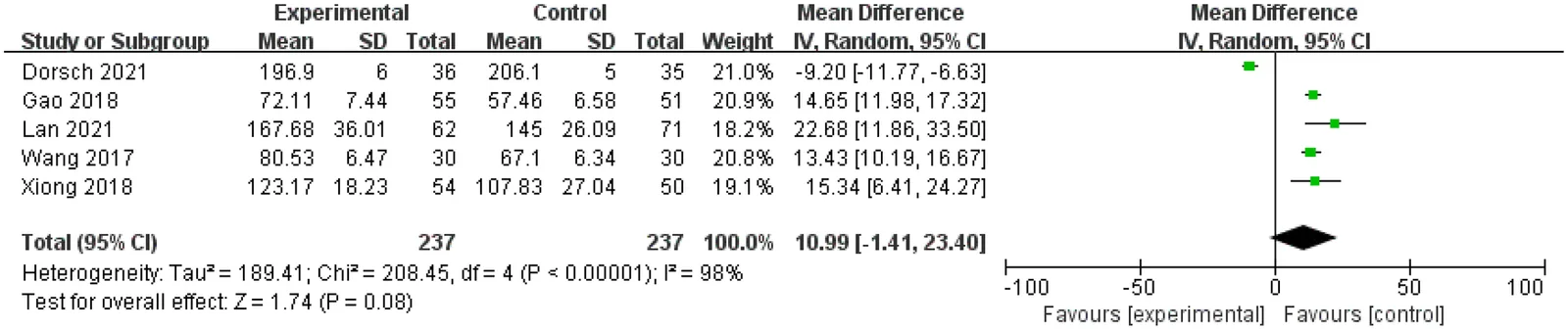
Forest plot of the effects of self-care of heart failure Index (SCHFI).

**Figure 8:**
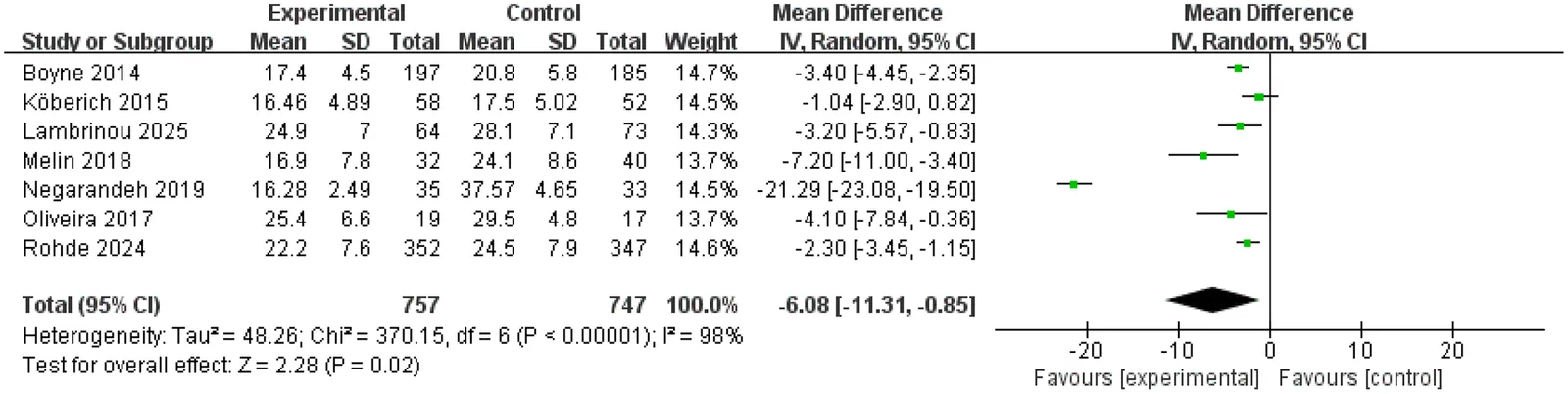
Forest plot of the effects of 9-European heart failure self-care behavior scale (9-EHFScBS).

**Figure 9:**
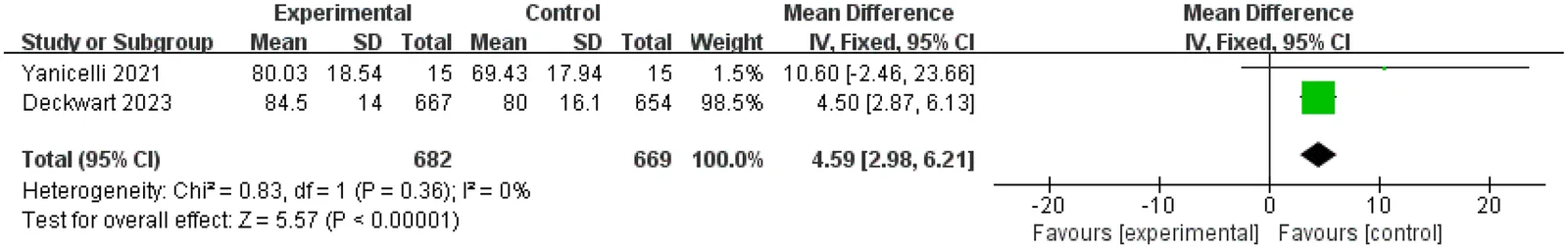
Forest plot of the effects of 9-EHFScBS (reverse coded).

A total of 18 studies reported self-care behavior data. Ten of these studies used the European Heart Failure Self-Care Behavior Scale (EHFSC) (58). Four of these studies used the EHFSC revised to a 9-item scale (9-EHFScBS) (59), 2 of them were reverse-scored and were therefore analyzed separately (53, 54), and 1 study had incomplete data (37). A lower score on the EHFSC indicates higher patient self-care ability. Seven of these studies used the Self-care of Heart Failure Index (SCHFI), which comprises three subscales (60). The total SCHFI scores could not be combined in two studies (40, 44), and finally five studies were included in the analysis. A higher score on the SCHFI scale indicates better self-care ability in patients with HF. Only one study used the Self-Management Scale for Heart Failure Patients (SMSHF) (61) to measure self-care ability and was therefore not included in the meta-analysis. The analysis indicated that patients in the nurse-led mHealth intervention group had significantly improved self-care behaviors compared with the control group when measured using 9-EHFScBS (MD: −6.08, 95% CI: −11.31 to −0.85, *P* = 0.02; Heterogeneity *I^2^* = 98%) (Figure 8) and reverse-scored 9-EHFScBS (MD: 4.59, 95% CI: 2.98 to 6.21, *P* < 0.00001; Heterogeneity *I^2^* = 0%) (Figure 9). However, no significant improvement in self-care behaviors was observed when measured using the SCHFI (MD: 10.99, 95% CI: −1.41 to 23.40, *P* = 0.08), with significantly high heterogeneity among 5 studies (*P* < 0.00001; *I^2^* = 98%) (Figure 7).

### 3.6 Secondary outcomes

#### 3.6.1 Effects of mHealth interventions on quality of life

**Figure 10:**
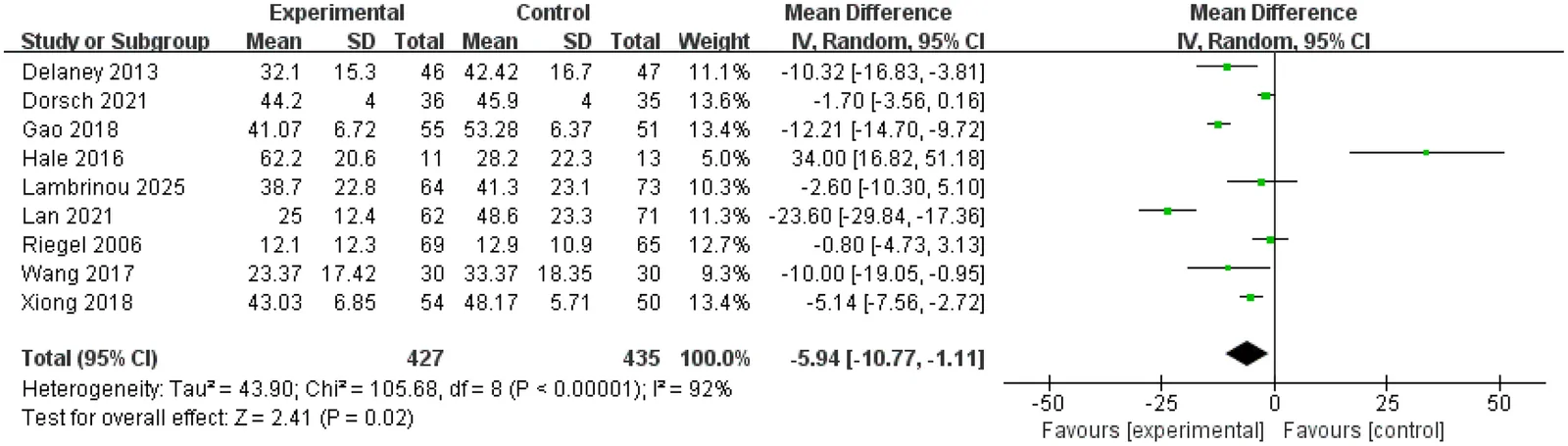
Forest plot of the effects of MLHFQ.

**Figure 11:**
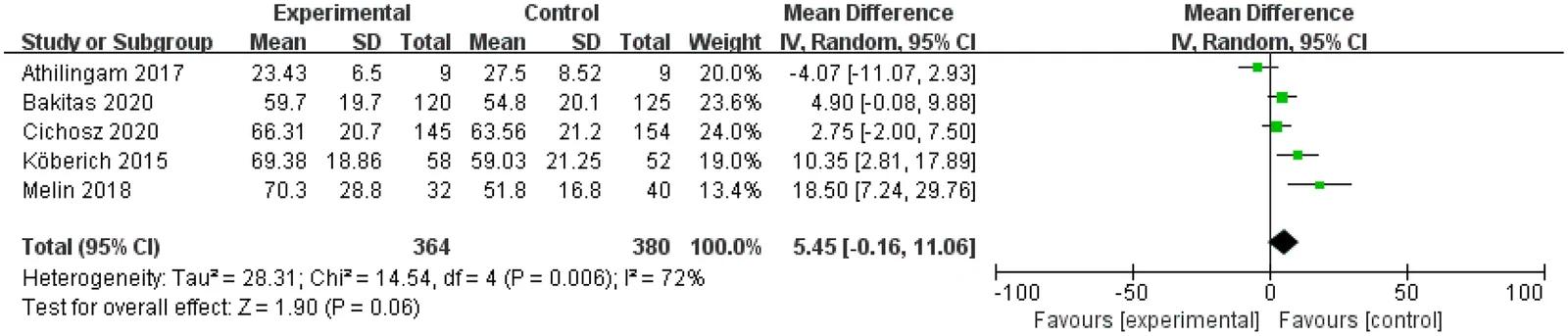
Forest plot of the effects of KCCQ.

**Figure 12:**
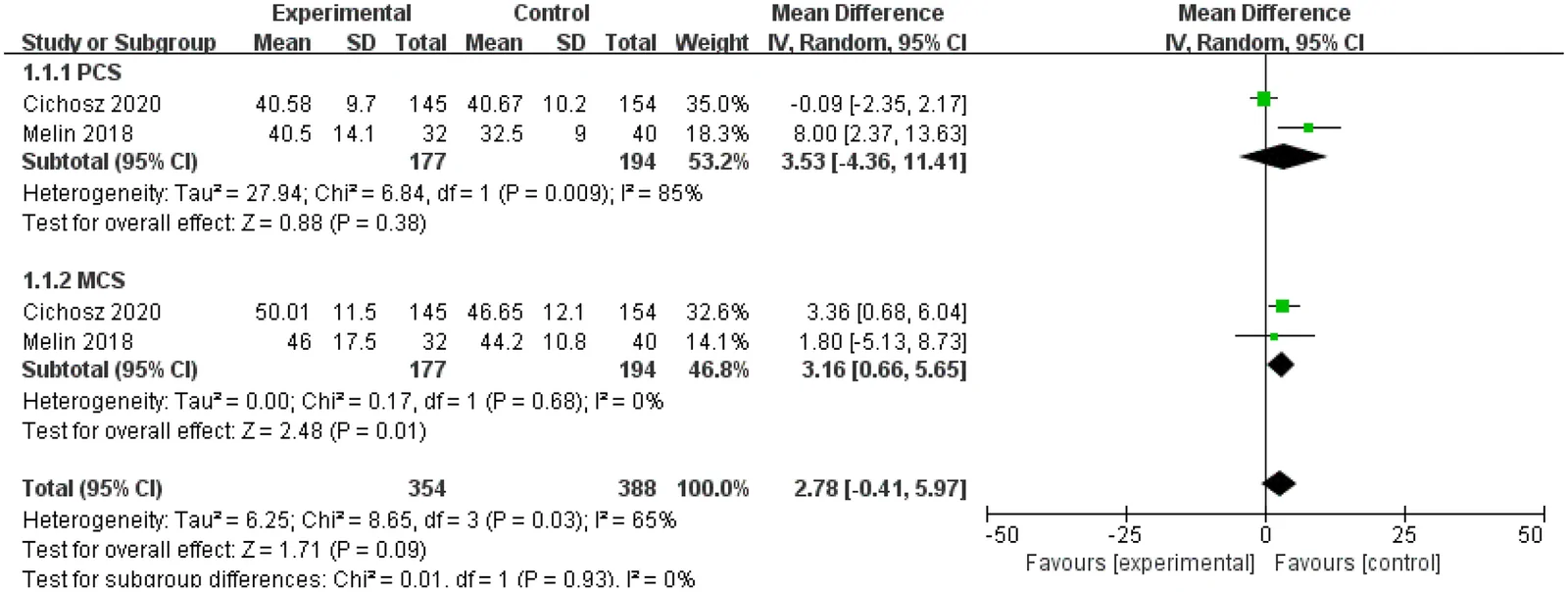
Forest plot of the effects of SF-36.

A total of 16 studies reported general quality of life data. Six of these studies used the HF-specific 23-item Kansas City Cardiomyopathy Questionnaire (KCCQ) (62), in which higher scores indicate better quality of life, but one study was excluded due to incomplete data (52). Nine of these studies used the Minnesota Living with Heart Failure Questionnaire (MLHFQ) (63), in which lower scores indicate a higher quality of life. Three of these studies used the 36-item Short-form Health Survey (SF-36) (64), in which higher scores indicate better quality of life, but one study was excluded due to incomplete data (50). The analysis indicated that quality of life was significantly improved in the nurse-led mHealth intervention group compared with the control group when measured by the MLHFQ (MD: −5.94, 95% CI: −10.77 to −1.11, *P* = 0.02; Heterogeneity *I^2^* = 92%) (Figure 10). However, when assessed using the KCCQ (MD: 5.45, 95% CI: −0.16 to 11.06, *P* = 0.06; Heterogeneity *I^2^* = 72%) (Figure 11) and SF-36 (MD: 2.78, 95% CI: −0.41 to 5.97, *P* = 0.09; Heterogeneity *I^2^* = 65%) (Figure 12), there was no significant difference in quality of life between the nurse-led mHealth intervention group and the control group.

#### 3.6.2 Effects of mHealth interventions on heart failure knowledge

**Figure 13:**
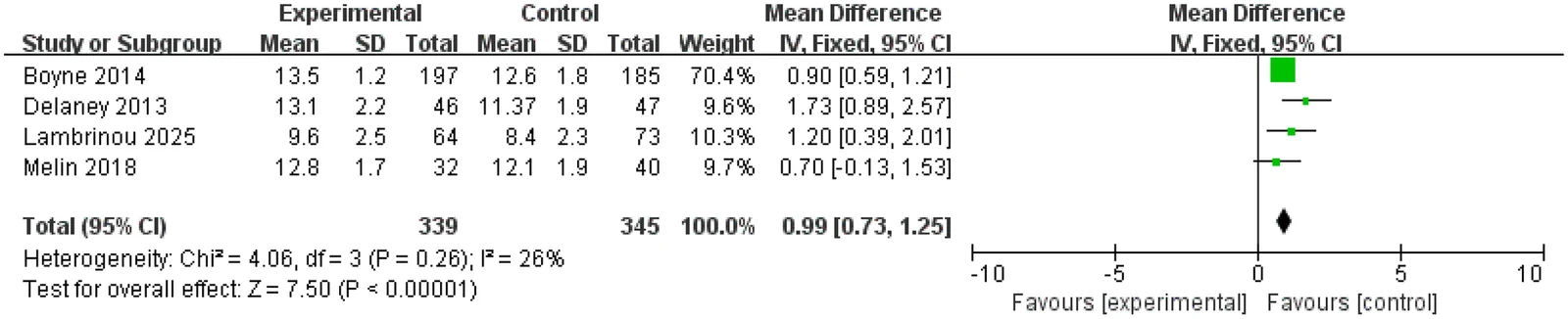
Forest plot of the effects of Dutch heart failure knowledge scale (DHFKS).

**Figure 14:**
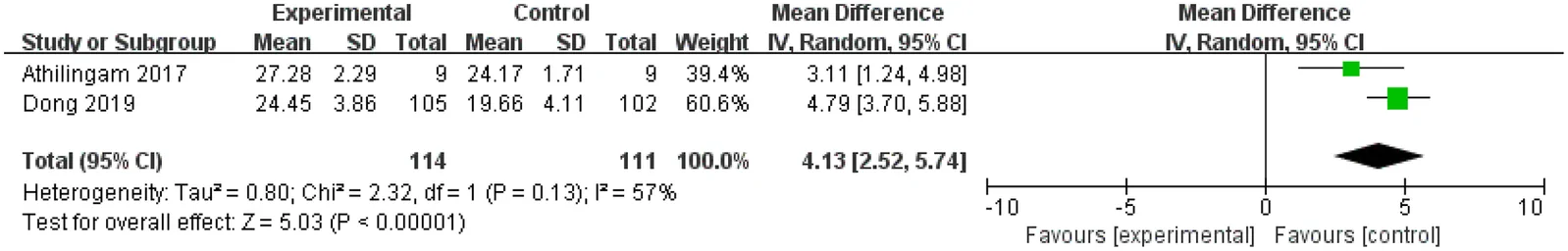
Forest plot of the effects of Atlanta heart failure knowledge test (AHFKT).

A total of 6 studies reported HF knowledge data. Among them, 4 used the Dutch Heart Failure Knowledge Scale (DHFKS) (65), and 2 used the Atlanta Heart Failure Knowledge Test (AHFKT) (66). Analyses were performed separately. Higher scores across both scales indicate that the patients have more knowledge of HF. The analysis indicated that when measured using the DHFKS, heart failure knowledge was significantly higher in the nurse-led mHealth intervention group compared with the control group (MD: 0.99, 95% CI: 0.73 to 1.25, *P* < 0.00001; Heterogeneity *I^2^* = 26%) (Figure 13). Consistent findings were observed with the AHFKT, with the nurse-led mHealth intervention group also showing significantly improved heart failure knowledge. (MD: 4.13, 95% CI: 2.52 to 5.74, *P* < 0.00001; Heterogeneity *I^2^* = 57%) (Figure 14).

#### 3.6.3 Effects of mHealth interventions on medication adherence

**Figure 15:**
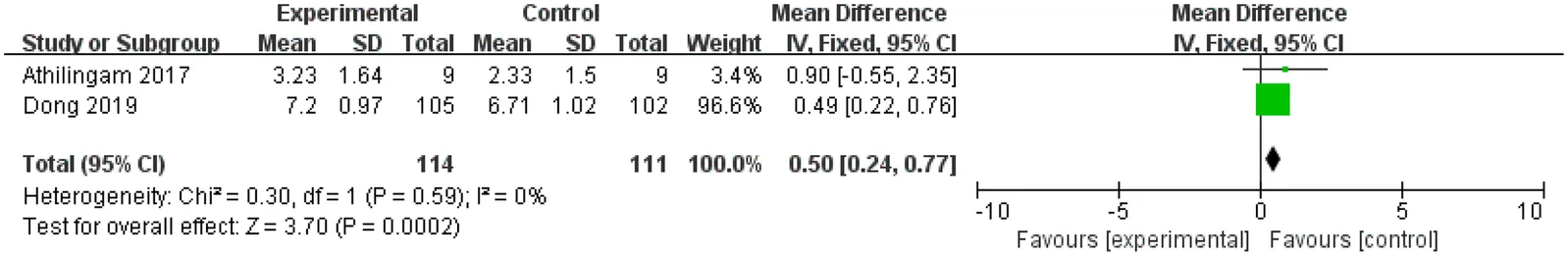
Forest plot of the effects of morisky medication adherence measure (MMAS).

Among the included studies, 3 investigated patients' medication adherence. Two of the studies using Morisky Medication Adherence Questionnaire (MMAS) (67) to assess patients' medication adherence were included in the meta-analysis, whereas one study using the Morisky Medication Adherence Scale (MMS) (68) was excluded due to an insufficient number of studies. A higher score on the MMAS indicates better medication adherence in patients with HF. The analysis indicated that medication adherence was significantly improved in the nurse-led mHealth intervention group compared with the control group (MD: 0.50, 95% CI: 0.24 to 0.77, *P* = 0.0002), with no heterogeneity among 2 studies (*P* = 0.59; *I^2^* = 0%) (Figure 15).

#### 3.6.4 Effects of mHealth interventions on exercise capacity

**Figure 16:**
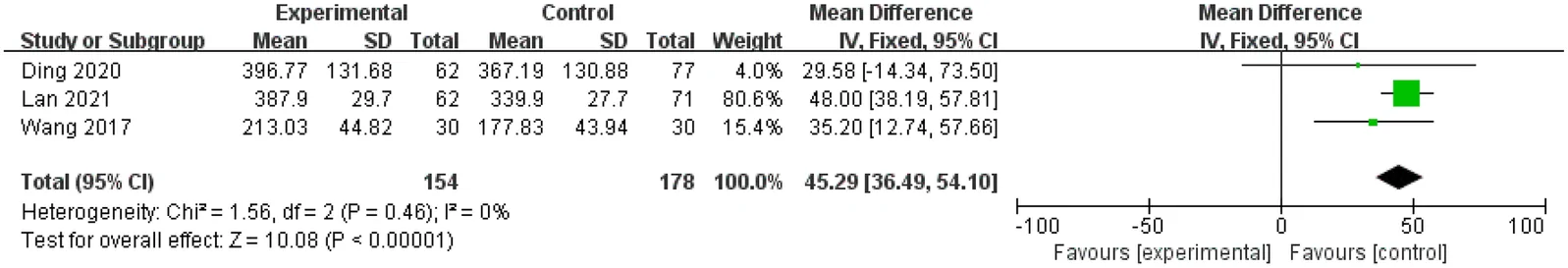
Forest plot of the effects of 6-minute walk test (6MWT).

Three studies measured exercise capacity in patients with HF, all using the 6-minute walk test (6MWT). The analysis indicated that cardiac function was significantly improved in the nurse-led mHealth intervention group compared with the control group (MD: 45.29, 95% CI: 36.49 to 54.10, *P* < 0.00001), with no heterogeneity among 3 studies (*P* = 0.46; *I^2^* = 0%) (Figure 16).

#### 3.6.5 Effects of mHealth interventions on mental Status

**Figure 17:**
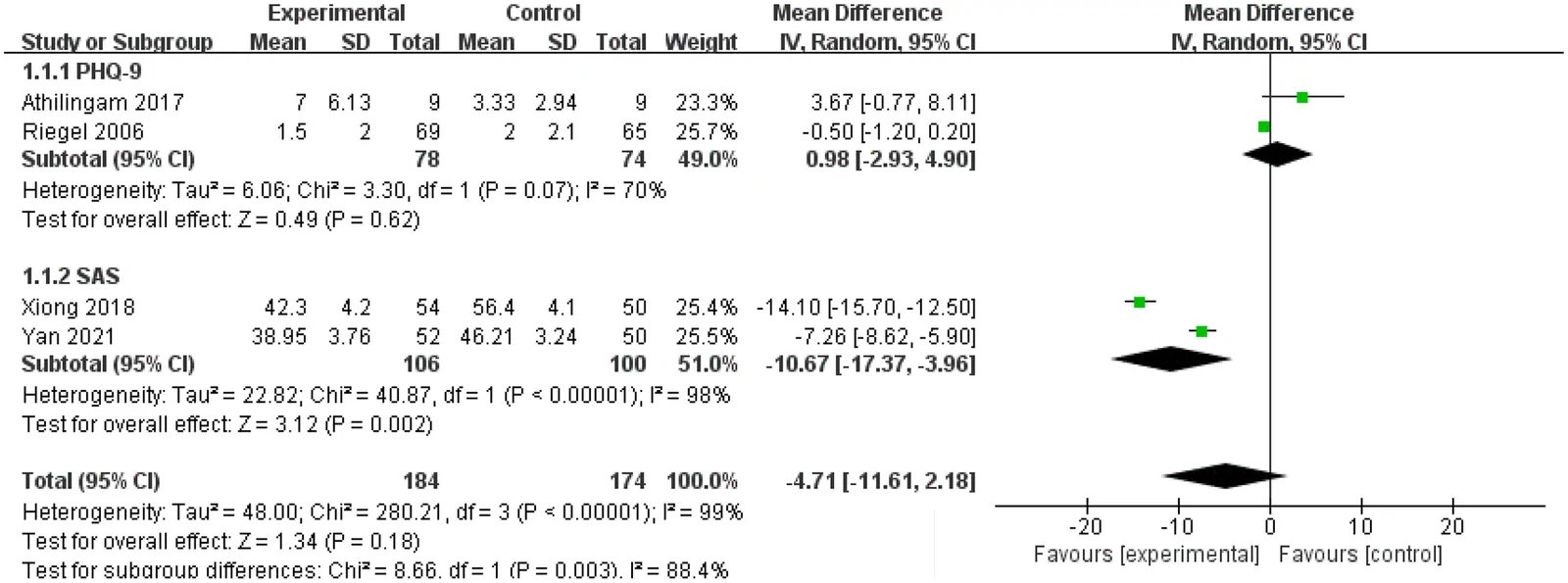
Forest plot of the effects of mHealth interventions on mental status.

A total of 7 studies reported mental status. Two studies measured depression using the Patient Health Questionnaire-9 (PHQ-9) (69), 2 studies measured anxiety using the Self-rating Anxiety Scale (SAS) (70), 1 study measured depression using the Patient Health Questionnaire-8 (PHQ-8) (71), 1 study measured depression using the Self-rating Depression Scale (SDS) (72), and 1 study measured depression using the Hamilton Depression Rating Scale (HAMD) (73). For all these scales, a higher total score generally indicated more severe symptoms. The analysis indicated that there was no significant difference in depression between the nurse-led mHealth intervention group and the control group when measured by the PHQ-9 (MD 0.98, 95% CI: −2.93 to 4.90, *P* = 0.62). However, anxiety was significantly improved in the nurse-led mHealth intervention group compared with the control group as measured by the SAS (MD −10.67, 95% CI: −17.37 to −3.96, *P* = 0.002). The combined results indicated that there was no significant difference between the nurse-led mHealth and usual-care groups (MD −4.71, 95% CI: −11.61 to −2.18, *P* = 0.18), with significantly high heterogeneity between studies (*P* < 0.00001; *I^2^* = 99%) (Figure 17).

### 3.7 Certainty of evidence

Supplementary Material Table S2 describes the summary of findings and evidence certainty evaluation. Certainty of evidence for both all-cause readmission and cardiovascular mortality was moderate, whereas for HF-related readmission and all-cause mortality was low. Certainty of evidence for HF self-care differed between applied tool, with high certainty level for 9-EHFScBS (reverse-coded), low for SCHFI and 9-EHFScBS. For secondary outcomes, the certainty of evidence was low for quality of life across all three applied tools (MLHFQ, KCCQ, and SF-36). For heart failure knowledge, the certainty of evidence was high when assessed using the DHFKS and low when assessed using the AHFKT. Certainty of evidence was moderate for medication adherence (MMAS) and moderate for exercise capacity (6MWT). For mental status, the certainty of evidence was low for both the PHQ-9 and the SAS.

### 3.8 Sensitivity analysis

**Figure 18:**
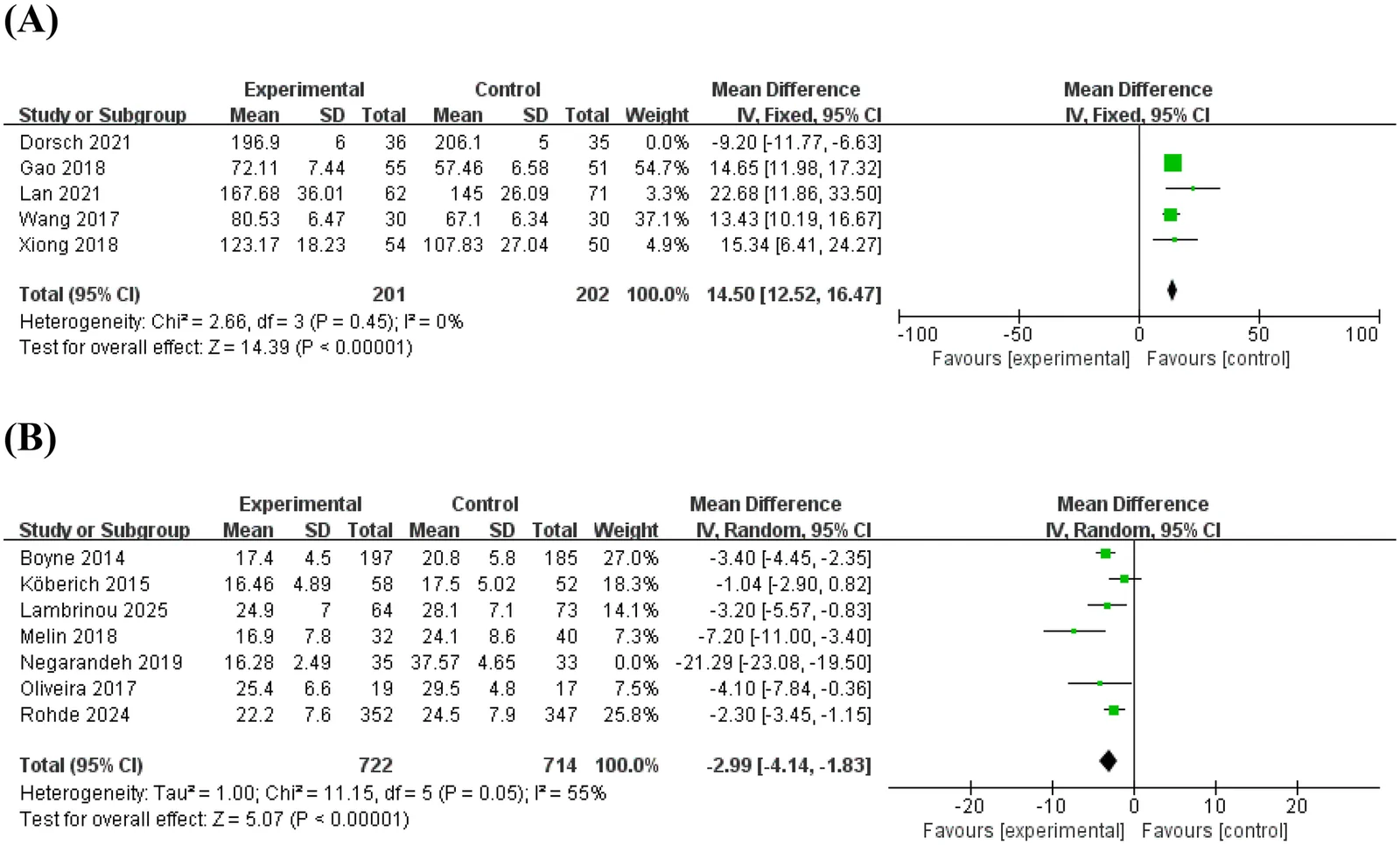
Sensitivity analysis. **(A)** Sensitivity Analysis of SCHFI. **(B)** Sensitivity Analysis of 9-EHFScBS.

Sensitivity analysis was conducted on outcomes that exhibited considerable heterogeneity (*P* < 0.01, *I^2^* > 50%), including self-care behaviors (SCHFI and 9-EHFScBS) and quality of life (MLHFQ and KCCQ), using the leave-one-out approach. Sensitivity analysis revealed high heterogeneity among the two studies due to differences in intervention methods and study quality (45, 51) (Figure 18). After removing one study, the SCHFI results were not only reversed but also heterogeneity decreased to 0. Further analysis indicated that the intervention duration in the two studies was relatively short, with interventions focusing on daily monitoring, and the control group received high-quality usual care. In addition, the frequency and duration of interventions varied across other studies, which may have contributed to increased heterogeneity. Only two studies contributed data for each of the following outcomes: SF-36 for quality of life, AHFKT for heart failure knowledge, and PHQ-9 and SAS for mental status. Heterogeneity was relatively high for these outcomes. Due to the small number of included studies (*n* = 2), we did not use sensitivity analyses, as such analyses would not provide stable or meaningful inferences and would largely reflect the results of individual studies.

### 3.9 Analysis of publication bias

**Figure 19:**
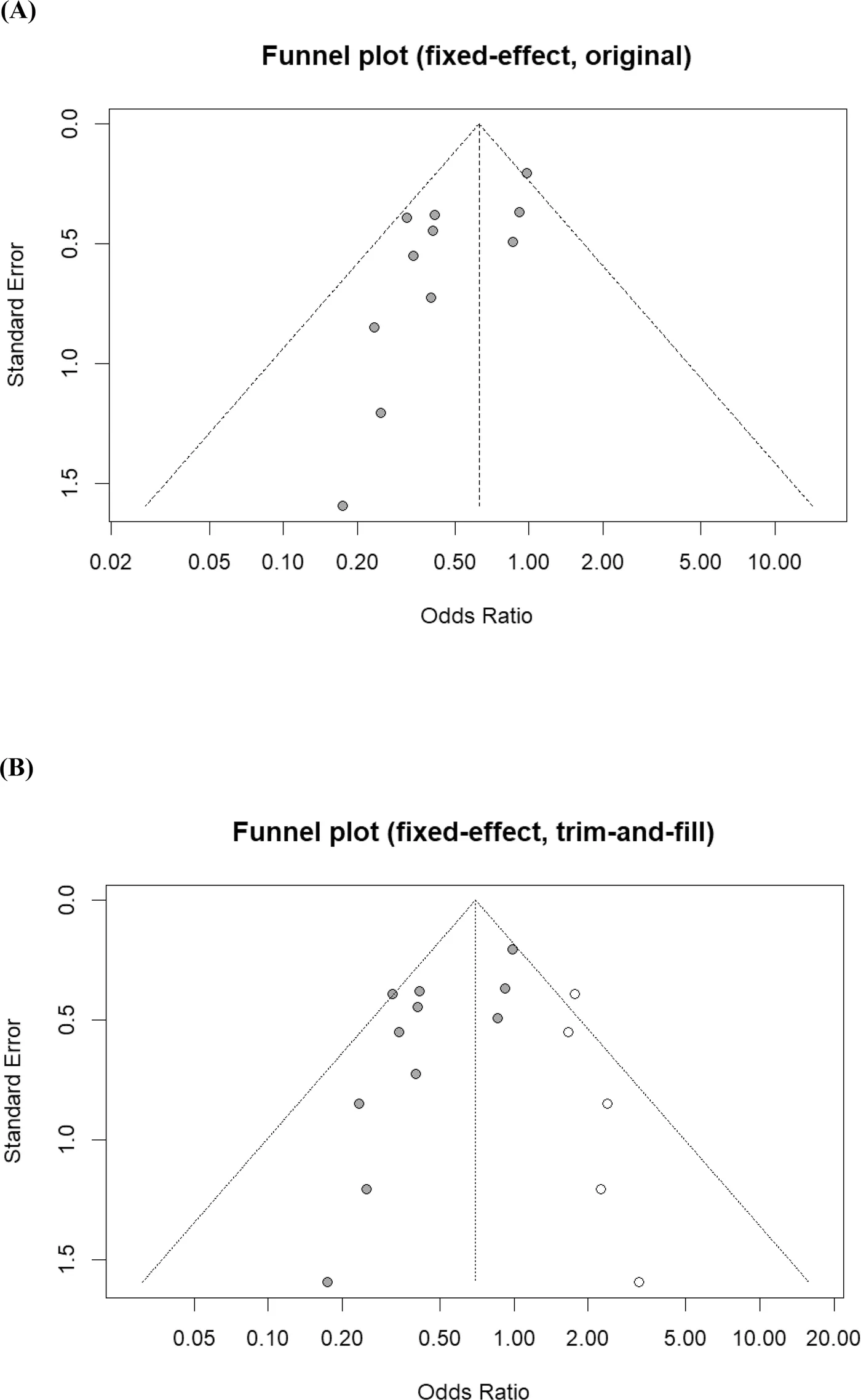

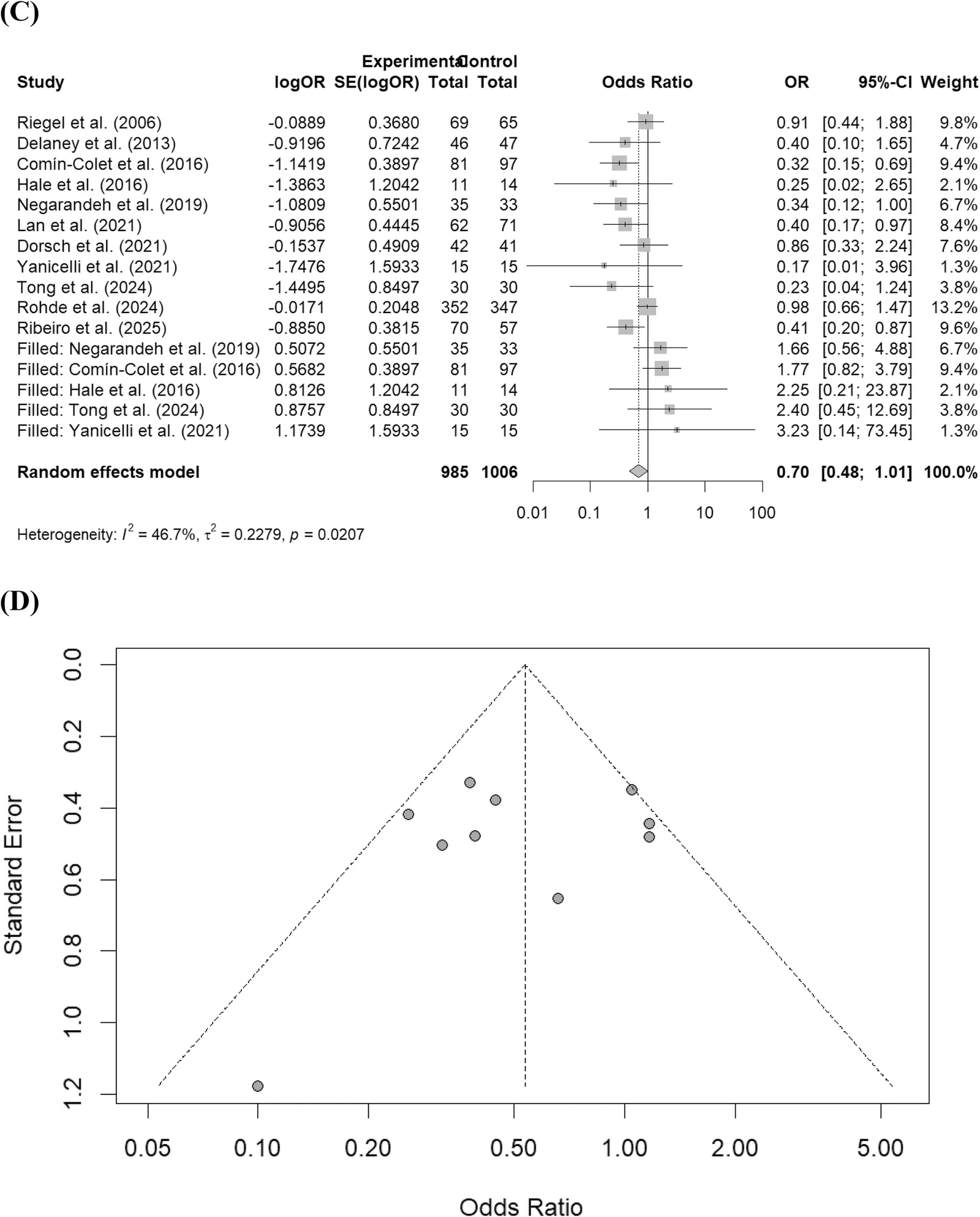
The funnel plots. **(A)** Funnel plots of HF-related readmission rate (original). Linear regression test of funnel plot asymmetry. Test result: t = −2.96, df = 9, *p*-value = 0.0159. **(B)** Funnel plots of HF-related readmission rate (trim-and-fill). Test result: t = −0.62, df = 14, *p*-value = 0.5459. **(C)** Forest plot of HF-related readmission rate (trim-and-fill adjusted). **(D)** Funnel plots of all-cause readmission rate. Linear regression test of funnel plot asymmetry. Test result: t = −0.64, df = 8, *p*-value = 0.5383.

The presence of publication bias was assessed for HF-related readmission and all-cause readmission using funnel plots and the Egger test. No publication bias was detected for all-cause readmission. In contrast, potential publication bias was suggested for HF-related readmission. After adjustment using the trim-and-fill method, the pooled effect estimate remained statistically significant and was materially unchanged. This suggests that the impact of publication bias on the overall pooled effect is limited, and the study conclusions are generally robust. The funnel plots and linear regression test results of funnel plot asymmetry are shown in Figure 19.

## 4 Discussion

### 4.1 Main findings

Given the limited nursing resources in hospital settings and the long recovery period of patients with heart failure, nurse-led telecare appears to be a promising long-term approach to providing continuous care for patients with HF (2, 74). In this meta-analysis, it was found that nurse-led mHealth interventions provide significant benefits for patients with HF. These programs were associated with improved patient self-care behaviors and medication adherence, along with better clinical and psychosocial outcomes: patients in nurse-led mHealth groups often achieved higher quality of life and self-care ability, fewer hospital readmissions, and even a lower risk of mortality in some analyses. For example, one recent meta-analysis showed that mHealth interventions significantly enhanced self-care and quality of life while decreasing healthcare costs (75). Overall, our findings align with prior studies and suggest that nurse-led mHealth can be an effective alternative or adjunct to traditional face-to-face management of HF.

### 4.2 Nurse-led intervention mechanisms

Nurse-led mHealth interventions leverage the unique advantages of nursing care through digital platforms. In these programs, nurses act as case managers and health coaches, using mobile technology to provide individualized health education, counseling, and monitoring for patients with HF. Common intervention components include tailored self-care guidance such as dietary adjustment, medication management, and symptom monitoring. It can not only remote tracking of patients' vital signs or symptoms, but also provide timely feedback and adjust care plans based on the monitored data (55, 57). Nurses typically communicate with patients regularly via telephone, text messages, or app messages to strengthen patients' adherence to treatment regimens and promptly address emerging health issues (21, 43, 56). This consistent support overcomes geographical barriers and maintains sustained contact between patients and healthcare providers (76). Through mHealth, nurses can closely monitor patients' daily conditions and intervene early, which helps meet the long-term management needs of patients with heart failure in real-life settings.

A key mechanism by which these interventions improve outcomes is patient empowerment. Nurse-led digital programs focus on developing skills and confidence, enabling patients to take a more active role in managing their condition. Nurses teach patients essential self-care tasks, such as daily weight monitoring, recognizing changes in symptoms, adhering to a low-sodium diet, and taking medications on time. Then nurses reinforce these behaviors through regular remote interactions (77). Guidance provided through user-friendly applications or telephone consultations helps patients internalize healthy habits and develop problem-solving skills when issues arise (40). In addition, when patients report worrying signs, nurses can liaise with physicians or other healthcare providers to coordinate care, ensuring that patients receive timely medical attention when needed. Through these processes, nurse-led mHealth interventions form a safety net for HF patients, promoting self-efficacy and reassuring them that professional support is always available. This combination of technology and nursing support underpins the effectiveness of the intervention, as patients feel continuously supported in the daily management of heart failure.

### 4.3 Comparison with usual care

These studies have shown that nurse-led mHealth interventions are comparable to traditional face-to-face heart failure management in effectiveness, and even superior in some aspects. For example, a meta-analysis of nurse-led tele-rehabilitation found that cardiac patients under remote management had significantly higher quality-of-life scores and self-care levels compared with those receiving traditional face-to-face rehabilitation (78). Key components of HF care, such as regular monitoring, timely medication adjustment, and patient education, can be efficiently delivered by technology. Core components of the chronic care model, including real-time communication between nurses and patients, self-management support, and structured follow-up, have been effectively replicated in telehealth settings (79). This suggests that nursed-led mHealth interventions can retain the key effective components of usual care, enabling patients to receive comprehensive disease management even without attending in-person hospital visits.

Notably, nurse-led mHealth interventions may offer advantages over usual care in certain aspects, as they provide more frequent contact and proactive management. Unlike intermittent outpatient follow-up, mHealth tools enable ongoing symptom monitoring and rapid responses to patient needs, with the potential to prevent complications. Several studies show that early detection of warning signs through remote monitoring can avoid some hospitalizations that might occur with less frequent follow-up (80). However, not all outcomes differ significantly between nurse-led mHealth interventions and usual care. A recent study indicated that tele-rehabilitation offered no significant advantage over face-to-face rehabilitation in reducing anxiety and depression among patients (78). This suggests that although nurse-led mHealth is generally equivalent or more beneficial than usual care in most management domains, it should be regarded as a complementary approach rather than a complete replacement for usual care. Combining nurse-led mHealth with usual care to leverage their respective strengths may yield the optimal outcomes.

### 4.4 Current challenges

Despite its promising potential, nurse-led mHealth self-management interventions also face several challenges and limitations in practical implementation. One major issue is the digital divide (81). Not all patients with HF have adequate access to the required technologies or feel comfortable using them. Heart failure is common among older adults, who may lack smartphones, reliable internet access, or the digital literacy needed to use mHealth tools effectively (82). A recent US study (81) found that only about 43% of hospitalized HF patients were eligible for effective mHealth use. The remaining patients faced multiple barriers, such as not having a smartphone (accounting for 51% of non-users) or being unable to access the internet (about 80%). In addition, the cost of devices or data plans can be a barrier for some patients. This means that a substantial proportion of patients may be excluded if interventions rely entirely on digital approaches (83). If mHealth programs fail to accommodate patients with low eHealth literacy or limited resources, their benefits may not reach those most in need of care.

Another challenge lies in maintaining long-term engagement and adherence among patients with HF. Many patients are enthusiastic about using mHealth applications initially, but their usage frequency often declines gradually as novelty fades and tasks become diverse. Inconsistent patient engagement weakens the effectiveness of interventions. What's more, potential benefits diminish if patients stop interacting with the app, fail to enter data regularly, or no longer respond to contact from nurses. However, many studies do not rigorously report levels of patient engagement or factors influencing adherence. A scoping review showed that definitions and measures of mHealth engagement vary widely in HF research, with patient engagement rates ranging from 45% to 100% across studies and often decreasing over time (84). The lack of consistent standards and transparency in measuring and reporting patient engagement makes it difficult to identify which strategies best support long-term participation. Developing better methods to assess and promote sustained engagement represents a key issue that urgently needs to be addressed in future implementation.

Furthermore, the current evidence base has several limitations that affect our confidence in generalizing these findings. Existing trials and meta-analyses demonstrate substantial heterogeneity in intervention protocols, patient populations, and outcome measures, making it difficult to draw definitive conclusions (17, 85). Improvements in certain outcomes are inconsistent across studies. For instance, not all analyses show significant improvements in quality of life or knowledge levels with mHealth interventions. Several mental outcomes such as anxiety and depression often show no significant changes (32, 40). Many studies have relatively short follow-up periods, mostly ranging from 3 to 6 months. Therefore, the long-term sustainability of benefits remains unclear. Evidence on the economic value of nurse-led heart failure programmers remains limited. Only a small number of studies have reported cost-effectiveness-related analyses, making it difficult to determine whether these interventions generate net cost savings (86). In summary, these limitations indicate that more high-quality and long-term studies are needed to fully establish the value of nurse-led mHealth strategies.

### 4.5 Future directions

Looking ahead, emphasis should be placed on optimizing and expanding the implementation of nurse-led mHealth interventions, while addressing solutions to the challenges mentioned above. First, more rigorous studies with longer follow-up periods are needed to strengthen the evidence base. We recommend conducting large, multicenter randomized controlled trials with extended follow-up to evaluate the long-term effects of interventions on clinical outcomes and patient-reported outcomes. Such studies should also strive to standardize outcome measures, including objective clinical endpoints and validated scales for self-care or quality of life, to facilitate comparison across trials and support robust meta-analyses. Ensuring diversity in patient populations within these trials will contribute to improving the generalizability of the findings.

Furthermore, strategies should be developed at the practical level to address barriers to accessibility and engagement. Nurse-led mHealth programs should be designed with user-friendly interfaces and adapted content to accommodate older patients or those unfamiliar with digital technologies. Early training and technical support can improve user confidence and acceptance. Healthcare systems and policymakers can consider initiatives to bridge the digital divide, such as providing device loans for patients who cannot afford the associated costs. In addition, developers should incorporate features that promote sustained engagement in applications, such as regular automated reminders, gamified progress tracking, or encouraging family member involvement, to support long-term self-management among HF patients.

## 5 Limitations

Although this systematic review and related meta-analysis indicated that nurse-led mHealth-based self-care interventions benefit patients with HF, this study has several limitations. First of all, the included studies differed in participant demographics, sample sizes, assessment tools, mHealth intervention durations, and types of interventions, which may lead to heterogeneity and bias in the results. Second, most included studies used self-reported measures and scales to assess outcomes, which may introduce bias in self-reported data. Third, not all studies contributed to each outcome analysis, which may affect the pooled results and increase heterogeneity. Therefore, the findings of this systematic review should be interpreted with caution. Finally, this review did not include grey literature.

## 6 Conclusion

In this systematic review, we provide a comprehensive overview of nurse-led mHealth self-management for patients with HF. This approach significantly improved readmission rates, self-care ability, and medication adherence among HF patients, but showed no advantage over usual care in terms of anxiety, depression, or mortality. These results highlight the potential of nurse-led mHealth strategies as an adjunct to usual care. Health policymakers should support and promote nurse-led digital self-management programs to fully leverage nurses' critical role in coordinating individualized HF care. Future research should focus on high-quality studies of cost-effectiveness and long-term outcomes, as well as exploring optimal implementation strategies to refine such interventions.

## Data Availability

The original contributions presented in the study are included in the article/Supplementary Material, further inquiries can be directed to the corresponding author.
