# Supplementary material for: Effectiveness of nurse-led mHealth-based self-care management for patients with heart failure: a systematic review and meta-analysis
Source: Front Cardiovasc Med. 2026 Sep 1;13:1882692. doi: 10.3389/fcvm.2026.1882692 (PMC13574621; doi:10.3389/fcvm.2026.1882692)
Supplement: Supplementary file 2 [file Table3.docx]

**Table 3 Certainty of Evidence**

| **Certainty assessment** | | | | | | | **№ of patients** | | **Effect** | | **Certainty** | **Importance** |
| --- | --- | --- | --- | --- | --- | --- | --- | --- | --- | --- | --- | --- |
| **№ of studies** | **Study design** | **Risk of bias** | **Inconsistency** | **Indirectness** | **Imprecision** | **Other considerations** | **nurse-led mHealth-based self-care management programs** | **usual care** | **Relative (95% CI)** | **Absolute (95% CI)** |  |  |
| **Heart Failure-related Readmission** | | | | | | | | | | | | |
| 11 | randomized trials | serious^a^ | not serious | not serious | not serious | publication bias strongly suspected^b^ | 141/813 (17.3%) | 204/817 (25.0%) | **OR 0.61** (0.48 to 0.78) | **81 fewer per 1,000** (from 112 fewer to 44 fewer) | ⨁⨁◯◯ Low^a,b^ |  |
| **All-cause Readmission** | | | | | | | | | | | | |
| 10 | randomized trials | serious^a^ | not serious | not serious | not serious | none | 149/490 (30.4%) | 219/492 (44.5%) | **OR 0.53** (0.41 to 0.69) | **147 fewer per 1,000** (from 198 fewer to 89 fewer) | ⨁⨁⨁◯ Moderate^a^ |  |
| **Cardiovascular Mortality** | | | | | | | | | | | | |
| 2 | randomized trials | not serious | not serious | not serious | serious^c^ | none | 38/433 (8.8%) | 42/444 (9.5%) | **OR 0.91** (0.58 to 1.45) | **8 fewer per 1,000** (from 37 fewer to 37 more) | ⨁⨁⨁◯ Moderate^c^ |  |
| **All-cause Mortality** | | | | | | | | | | | | |
| 6 | randomized trials | serious^a^ | not serious | not serious | serious^c^ | none | 65/652 (10.0%) | 86/667 (12.9%) | **OR 0.74** (0.52 to 1.04) | **30 fewer per 1,000** (from 57 fewer to 4 more) | ⨁⨁◯◯ Low^a,c^ |  |
| **Heart Failure Self-Care (assessed with: SCHFI)** | | | | | | | | | | | | |
| 5 | randomized trials | serious^a^ | not serious | not serious | serious^c^ | none | 237 | 237 | - | MD **10.99 higher** (1.41 lower to 23.4 higher) | ⨁⨁◯◯ Low^a,c^ |  |
| **Heart Failure Self-Care (assessed with: 9-EHFScBS)** | | | | | | | | | | | | |
| 7 | randomized trials | serious^a^ | serious^d^ | not serious | not serious | none | 757 | 747 | - | MD **6.08 lower** (11.31 lower to 0.85 lower) | ⨁⨁◯◯ Low^a,d^ |  |
| **Heart Failure Self-Care (assessed with: 9-EHFScBS) (reverse-coded)** | | | | | | | | | | | | |
| 2 | randomized trials | not serious | not serious | not serious | not serious | none | 682 | 669 | - | MD **4.59 higher** (2.98 higher to 6.21 higher) | ⨁⨁⨁⨁ High |  |
| **Quality of Life (assessed with: MLHFQ)** | | | | | | | | | | | | |
| 9 | randomized trials | serious^a^ | serious^d^ | not serious | not serious | none | 427 | 435 | - | MD **5.94 lower** (10.77 lower to 1.11 lower) | ⨁⨁◯◯ Low^a,d^ |  |
| **Quality of Life (assessed with: KCCQ)** | | | | | | | | | | | | |
| 5 | randomized trials | serious^a^ | not serious | not serious | serious^c^ | none | 364 | 380 | - | MD **5.45 higher** (0.16 lower to 11.06 higher) | ⨁⨁◯◯ Low^a,c^ |  |
| **Quality of Life (assessed with: SF-36)** | | | | | | | | | | | | |
| 2 | randomized trials | not serious | serious^d^ | not serious | serious^c^ | none | 354 | 388 | - | MD **2.78 higher** (0.41 lower to 5.97 higher) | ⨁⨁◯◯ Low^c,d^ |  |
| **Heart Failure Knowledge (assessed with: DHFKS)** | | | | | | | | | | | | |
| 4 | randomized trials | not serious | not serious | not serious | not serious | none | 339 | 345 | - | MD **0.99 higher** (0.73 higher to 1.25 higher) | ⨁⨁⨁⨁ High |  |
| **Heart Failure Knowledge (assessed with: AHFKT)** | | | | | | | | | | | | |
| 2 | randomized trials | serious^a^ | serious^d^ | not serious | not serious | none | 114 | 111 | - | MD **4.13 higher** (2.52 higher to 5.74 higher) | ⨁⨁◯◯ Low^a,d^ |  |
| **Medication Adherence (assessed with: MMAS)** | | | | | | | | | | | | |
| 2 | randomized trials | serious^a^ | not serious | not serious | not serious | none | 114 | 111 | - | MD **0.5 higher** (0.24 higher to 0.77 higher) | ⨁⨁⨁◯ Moderate^a^ |  |
| **Exercise Capacity (assessed with: 6MWT）** | | | | | | | | | | | | |
| 3 | randomized trials | serious^a^ | not serious | not serious | not serious | none | 154 | 178 | - | MD **45.29 higher** (36.49 higher to 54.1 higher) | ⨁⨁⨁◯ Moderate^a^ |  |
| **Mental Status (assessed with: PHQ-9)** | | | | | | | | | | | | |
| 2 | randomized trials | not serious | serious^d^ | not serious | serious^c^ | none | 78 | 74 | - | MD **0.98 higher** (2.93 lower to 4.9 higher) | ⨁⨁◯◯ Low^c,d^ |  |
| **Mental Status (assessed with: SAS)** | | | | | | | | | | | | |
| 2 | randomized trials | serious^a^ | serious^d^ | not serious | not serious | none | 106 | 100 | - | MD **10.67 lower** (17.37 lower to 3.96 lower) | ⨁⨁◯◯ Low^a,d^ |  |

**CI:** confidence interval; **MD:** mean difference; **OR:** odds ratio

#### Explanations

a. Allocation concealment was not described in some articles.

b. Egger's test indicated the presence of publication bias.

c. The pooled 95% confidence interval (CI) encompasses both "no effect" and "significant benefit".

d. High heterogeneity
